# The Relationship between Sleep and Cognitive Performance in Autism Spectrum Disorder (ASD): A Pilot Study

**DOI:** 10.3390/children5110153

**Published:** 2018-11-16

**Authors:** Nouf Backer Al Backer, Malak Alzawad, Hafiz Habibullah, Shahid Bashir

**Affiliations:** 1 Department of Pediatric, King Saud University Medical City, Faculty of Medicine, King Saud University, Riyadh P.O. 11461, Saudi Arabia; nalbacker@ksu.edu.sa; 2 Neuroscience Center, King Fahad Specialist Hospital Dammam, Dammam P.O. 15215, Saudi Arabia; malakjz1@gmail.com (M.A.); Hafiz.habibullah@kfsh.med.sa (H.H.)

**Keywords:** cognitive function, sleep, autism, attention

## Abstract

Background: Sleep concerns are common in children with autism spectrum disorders (ASD). The impact of poor sleep on cognitive performance in ASD children is not well-established. We investigated the possible correlation between sleep quality in ASD children and cognitive performance. The Cambridge Neuropsychological Test Automated Battery (CANTAB) was administered to examine specific components of non-verbal cognition. Methods: The Children’s Sleep Habits Questionnaire (CSHQ) and actigraphy-measured data from 18 children with diagnosis of ASD were evaluated. Motor planning task (MOT), simple reaction time task (SRT) and the intradimensional/extradimensional shift (IED) of CANTAB were administered. Results: ASD good sleeper (ASD-GS) showed significant better response time for SRT task as compared to ASD poor sleeper (ASD-PS) based on CSHQ score. Parameters of bedtime resistance (r = 0.531, *p* = 0.023), sleep anxiety (r = 0.474, *p* = 0.047) from CSHQ and actigrapgy dependent (wake after sleep onset (WASO) (r = 0.430, *p* = 0.024) were significantly correlate with response time of SRT task. Conclusion: We conclude that some signs reflecting the presence of poor sleep in ASD correlate with various aspects of motor output on non-verbal performance tasks. The question is raised whether poor sleep in non-complaining persons with autism should be treated.

## 1. Introduction

Autism spectrum disorder (ASD) is characterized by impairments in social communication and social interaction as well as the presence of restricted and repetitive behavioral patterns (American Psychiatric Association, 2013) [1]. Sleep disorders are common associated conditions, with a prevalence estimated to range from 44–83% [2,3].

Defining sleep status can be challenging in study populations such as children with ASD who have limited sleep-related data available. Epidemiological data on sleep behaviors and sleep quality in individuals with ASD have been largely derived from parent report. The majority of sleep-related information available in the large, public ASD databases is limited to the Children’s Sleep Habits Questionnaire (CSHQ) [4]. The CSHQ, a widely used and validated instrument, is based on parent report and may differ from objective measures of a sleep disturbance in a child with ASD.

Objective measures used to define sleep concerns include polysomnography (PSG) and actigraphy. PSG Abnormalities provides evidence of sleep disorder, eliminating possible parental over reporting of sleep concerns [5].

However, some children with ASD may not be able to tolerate PSG, given the anxiety of “sleeping with wires” in a non-home environment, intolerance due to a given child’s tactile sensitivities, or both. Furthermore, PSG is a costly methodology and the limited amount of information provided by a pediatric PSG study may not justify its large expense. Actigraphy, a modality that measures sleep and wake patterns based on limb movement, is less-intrusive and costly than PSG, and can be performed in a child’s home setting. However, studies have shown mixed results in using actigraphy to define parental sleep concerns in ASD, with most (but not all) investigators documenting a mismatch between parent report and actigraphic measures [6,7,8].

Based on actigraphy data and parental questionnaires, the sleep problems in ASD patients can be best characterized as sleep onset and maintenance insomnia [9]. ASD patients have difficulty to maintain the required latency time which commonly presented as difficulty to initiate and/or maintain sleep, waking during the night and reduced total sleep [10,11,12]. It is well known that poor sleep quality in typically developing children might result in behavior disturbance, attention deficits [13,14] hyperactivity and poor cognition [15]. Sleep disturbances in ASD patients might interfere with their attention, learning and impulsivity [16], but less is known about the effects of sleep problems on day-time cognitive and adaptive performance in ASD patients. It was hypothesized that the presence of sleep disorders could negatively influence intrinsic ASD characteristics, especially with regard to cognition and behavior [10,17,18].

Although cognition is known to be affected by sleep quality in typically developing children [19,20,21,22,23], there is a paucity of data to establish such relationship among children with ASD. In one study, IQ scores for children with ASD were correlated with subjective sleep quality and quantity, though causal relationships remain unclear [24].

In regard to pattern of attention in ASD, sustained attention has been preserved in most studies [25,26]. In regard to cognitive function, the pattern of memory performance in high-functioning ASD, intact verbal working memory contrasts with growing evidence of abnormal visuo-spatial working memory [27,28,29] published the only study on motor procedural memory in children with high-functioning ASD, reporting slower motor procedural learning in a serial reaction time (RT) task.

The computer-controlled system known as the Cambridge Neuropsychological Test Automated Battery (CANTAB) is a nonverbal (visually presented) set of tasks developed to recognize areas of the brain for cognition [25,26,27,28,29,30,31].

Three CANTAB subtests, the Motor task, simple reaction time, and the Intradimensional/Extradimensional (IED) tasks, test the functions of prefrontal cortex region of the brain which contains planning and programming areas of the human brain. The neuroimaging data has provided support in exploring their roles [27].

The present study aimed to investigate the correlation between whether poor sleep (determine by parent-reported domains on the CSHQ and actigraphy in ASD patients, correlates with non-verbal cognitive performance. To avoid performance variability due to test modality, only non-verbal tasks were used.

This study provided pilot data toward a new study, in which we intend to expand the sample and apply some strategies to improve sleep quality in ASD patients, then investigate the effect of good sleep quality on behavioral and cognitive parameters.

## 2. Material ad Methods

18 children ages between 7–10 (mean ± SD, 8.20 ± 1.70) years with a diagnosis of ASD who are coming for follow up in the clinic from the period of March 2016–March 2017 were recruited. Participants were diagnosed previously with ASD by a qualified professional using the gold standard measure to diagnose ASD, Autism diagnostic observation schedule (ADOS). All were non-verbal males. Intelligence quotient (IQ) for all autistic children was below 80.

Participants should have stable medical and behavioral conditions with no change in medications in the last 6 months. Participants were excluded from the study if they have a co-morbid psychiatric disorder, a significant vision or hearing loss, uncontrolled seizure in the last 6 months and a complex neurological disorder (e.g., cerebral palsy, tuberous sclerosis, neurofibromatosis, Rett disorder etc.).

The study was explained to the parent and informed consent was obtained. The study was approved by the local committee of King Saud University ((E-12-800, date of approval April 2014). Parents completed a series of questionnaires about sleep behavioral of their children and were given instructions on care of the actigraphy and how to keep a complete daily sleep diary. Participants were instructed to return actigraphy back after one week along with their sleep diary.

### 2.1. Sleep Measures

The Children’s Sleep Habits Questionnaire (CSHQ) is a validated parentally-completed questionnaire used to examine sleep behavior in toddlers, preschool and school-aged children with a variety of conditions, including ASD [5,32].

Subscales measure insomnia-related dimensions such as bedtime resistance, sleep anxiety, sleep onset delay, sleep duration, and night wakings, as well as other dimensions such as daytime sleepiness, sleep disordered breathing, and parasomnias. A total score can be calculated from all of the dimensions [5,32].

### 2.2. Actigraphy

Children were to undergo two consecutive weeks of actigraphy monitoring. The actigraph was maintained on the child’s wrist, as tolerated. Instrumentation—Actigraphy measurements were obtained with the use of AW-64 Actiwatch^®^ monitors (Philips Respironics, Bend, OR, USA). It was worn on the dominant wrist over seven consecutive 24-h periods. Actigraphy, widely used in sleep research and clinical practice, has been validated as a highly reliable method to differentiate sleep from wake [33] based on the detection of movement and rest. Each actigraphy watch contains an accelerometer, which is able to detect motion greater than 0.01 g-force in all directions, and translate it into an electrical signal. This information is subsequently stored in memory within the watch as activity counts, a unit that expresses the largest of all measured accelerations over a predefined measurement epoch.

Data from the actigraphs were downloaded to a personal computer where all sleep intervals were manually placed on an actogram, or visual representation of the actigraphy data. The total nighttime sleep duration (TST) was the sum of all sleep epochs within the interval between the time set on the actogram for nighttime sleep and morning wake time. Sleep efficiency (SE) was calculated as the ratio of total nighttime sleep duration to the total time in bed. Sleep latency (SL) was calculated as the time required for sleep onset after lights out (first attempt to go to sleep). Wake after sleep onset (WASO) was measured as the sum of all wake epochs during the sleep period and reflects the number of minutes scored as wake that exceeded the sensitivity threshold. The fragmentation index (FI), captures all movement regardless of the intensity of the movement. All sleep variables were calculated using Actiware V5 software (Philips Respironics, Bend, OR, USA).

### 2.3. CANTAB Testing

The three subtests from the CANTAB (http://www.cambridgecognition.com/cantab/) computerized battery were administered and responses were recorded directly with a touch-sensitive screen. Multiple training trials to learn the requirements of each task were given to each participant.

Before the actual test is started orientation trials were given to familiarize the subjects with the tests. The co-investigators were trained for CANTAB testing and they administered the tests to the participants in supervision of their consultant.

#### 2.3.1. Motor Screening Task (MOT)

MOT task provides an assessment (speed, accuracy and number of errors) involving the selection of colored crosses in different locations on the screen as quickly and accurately as possible by the participant.

#### 2.3.2. Intradimensional/Extradimensional (IED) Shift

IED is a test that assesses the shifting and flexibility of attention in the fronto-striatal areas of the brain and takes about 7 min. There are two dimensions that are used in this test: color-filled shapes and white lines. The simple stimuli are the color-filled shapes and the compound stimuli are both the color-filled shapes and the white lines. This test started with two simple stimuli appearing in the screen and the subject has to learn the correct stimuli and respond by touching it. Feedbacks teach the subject the correct stimuli. After six correct responses, the stimuli and/or the rule changes. IED test assesses the errors, and the number of trial and stages completed. The detail description of the task mentioned in CANTAB website (http://www.cambridgecognition.com/cantab/cognitive-tests/intra-extra-dimensional-set-shift-ied/).

### 2.4. Simple Reaction Time (SRT)

The SRT task measures simple reaction time, general alertness, and motor speed through delivery of a known stimulus to a known location to elicit a known response. The only uncertainty is regarding when the stimulus will occur, by having a variable interval between the trial response and the onset of the stimulus for the next trial.

The detail description of the task mentioned in CANTAB website (http://www.cambridgecognition.com/cantab/cognitive-tests/SRT).

### 2.5. Statistical Analysis

Analysis of the data were performed by Statistical Package for Social Sciences (SPSS) version 23 (SPSS Inc., IBM, Chicago, IL, USA).

We divided the sample into “poor sleepers” (upper quartile on the total score of the CSHQ-ASD) and “good sleepers” (lower quartile) for comparisons. Descriptive statistics were calculated for the entire sample as well as the two groups (ASD-poor sleepers; ASD-good sleepers). All statistical significance was set at *p* value <0.05. T tests and analysis of variance (ANO-VA) were used for continuous data generated from the CSHQ scores and actigraphy. A χ^2^ analysis was used for categorical data generated from the CSHQ, comprehensive sleep history questionnaire, and actigraphic data. The prevalence of sleep disturbances in the ASD and TD cohorts was analyzed using a *t* test for continuous data and a χ^2^ test for categorical data.

Spearman rank correlations (rs) were used to evaluate associations between actigraphic sleep variables, subjective sleep measures, and behavioral scales. Results are presented as mean (standard deviation) and given the pilot nature of the study, we did not pursue a formal multiple comparison adjustment.

## 3. Results

### 3.1. Sleep-Related Measures

Regarding demographic findings, no significant difference was found for age between groups (*p* > 0.05, Table 1). Table 1 summarizes sleep performance bases on CHSQ and actigraphy in ASD. The present subgroup of participants signs of poor sleep. All of the CSHQ scores were significantly different between groups (Table 1). Total score (*p* = 0.001), bedtime resistance (*p* = 0.001), sleep anxiety (*p* = 0.019), and night wakings (*p* = 0.007) subscores (CSHQ scale) were higher in the ASD-PS group when compared to the ASD-GS group.

For actigraphy variable the ASD-GS showed better score but did not reach significant level as compared to ASD-PS for Total Sleep Time (TST) (*p* = 0.083), Efficiency (*p* = 0.189), Wake After Sleep Onset (WASO) (*p* = 0.077) and Number of Awakenings (*p* = 0.692).

### 3.2. Cognitive Performance

**Figure 1:**
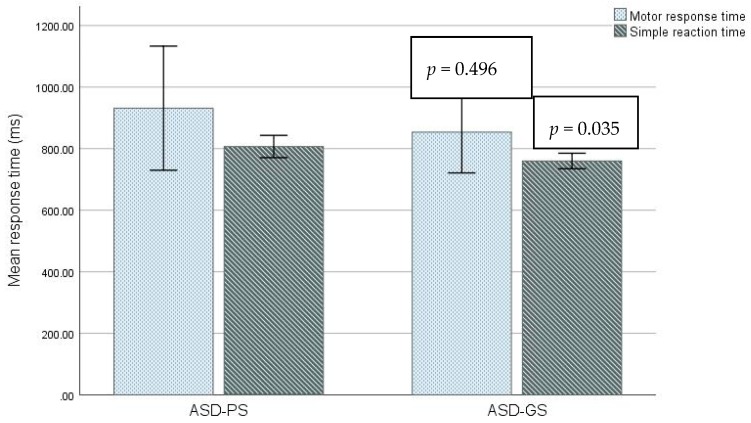
Comparison of cognitive function through response time in motor screening task and simple reaction time (ms) task among ASD poor sleepers (ASD-PS), ASD good sleepers (ASD-GS). The bars showed mean ± Std. D data.

Table 2 shows the results of neuropsychological battery scores for ASD in the two groups. ASD-PS had a longer SRT time than ASD-GS (806.9 ± 50.6 versus 759.8 ± 30.1, *p* = 0.035, Figure 1). The maximum time to react (SRT-Maximum) was not significantly longer in the ASD-PS group (799.8 ± 106.8) compared to the ASD-GS group (737.6 ± 27.6, *p* = 0.130, Table 2). The response time in MOT was not significantly longer for ASD-PS (931.4 ± 281.9) and ASD-GS (853.5 ± 158.4, *p* = 0.496, Table 2).

The IED, MOT mean error and SRT-Percentage were not significantly greater/longer among ASD-PS compared to ASD-GS (for values see Table 2).

### 3.3. Correlation between Sleep Parameters and Cognitive Functions

**Figure 2:**
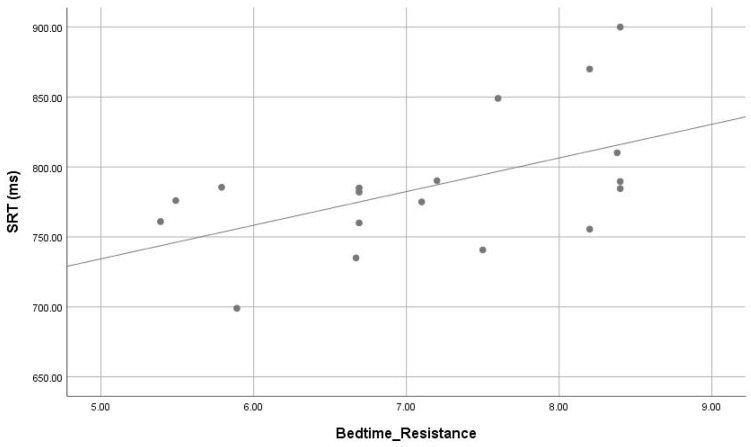
The positive correlation (r = 0.531, *p* = 0.023) of bedtime resistance (CSHQ subscale) and simple reaction time (ms) in ASD.

**Figure 3:**
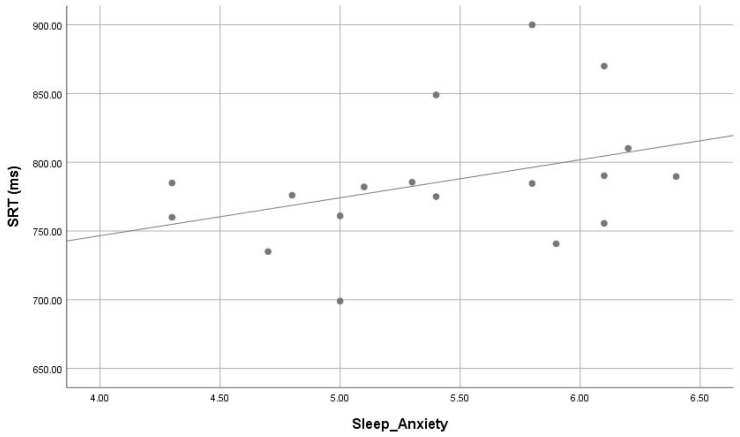
Correlation (r = 0.474, *p* = 0.047) of sleep anxiety (CSHQ subscale) and simple reaction time (ms) in ASD.

**Figure 4:**
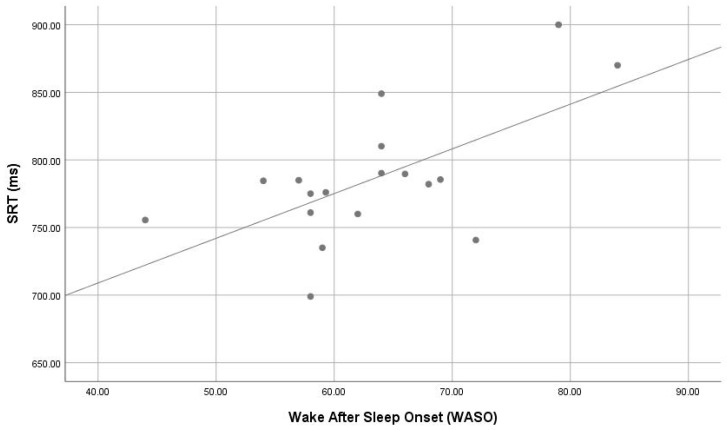
Correlation (r = 0.430, *p* = 0.024) of wake after sleep onset (actigrapgy subscale) and simple reaction time (ms) in ASD.

The correlations between sleep parameters of CSHQ and actigraphy dependent with cognitive variables are presented in Figure 2, Figure 3 and Figure 4. Parameters of poor sleep signs of poor sleep (i.e., bedtime resistance (r = 0.531, *p* = 0.023), and sleep anxiety (r = 0.474, *p* = 0.047) from CSHQ) were significantly correlate with response time of SRT task. The results indicate that sleep latency and wake after sleep onset percentage correlated positively motor speed response. There was significant correlation of actigrapgy dependent wake after sleep onset (WASO) (r = 0.430, *p* = 0.024) with response time of SRT task.

No significant correlation was observed between MOT and IED task in ASD and in the comparison group.

## 4. Discussion

Our results show that the ASD-PS group had a significantly higher score than that of the ASD-GS group for Children’s Sleep Habits Questionnaire (CSHQ) subscales and actigraphy variable. When considering the cognitive function motor response time in SRT task were significant between the two groups. The performance on the IED task was similar in both groups, but ASD-GS participants tended to make fewer errors. The findings demonstrate that, on the one hand, ASD-PS individuals are able to perform on memory and attention tasks at an accuracy level equivalent to that of the ASD-GS, even when they present signs of poor sleep. It is therefore plausible that certain aspects of sleep are important enough to result in reduced performance, at least on non-verbal tasks, in ASD. These results are consistent with the literature on performance in high-functioning ASD [33].

On the other hand, we found speed of processing to be slower than in the ASD-GS for most tasks. There are at least two possible explanations for that slowness. One possibility is that persons with ASD-GS use alternate strategies to perform at a level equal to, if not better than, the ASD-PS. Indeed, brain imaging studies have shown that persons with ASD engage alternate pathways in perceptual, attention and memory tasks to achieve at least normal levels of performance [34,35,36]. Another possibility is that slow speed of processing is the consequence of an alertness deficit.

Correlation between sleep and cognitive performances in ASD we expected to find, in this group, a significant correlation between signs of poor sleep, on the one hand, and response time, on the other hand. The fact that a significant correlation was observed between markers of poor sleep and typical performance (speed) but not with atypical performance (working memory and sustained attention) does not support an association between slow non-verbal processing and poor sleep in ASD. Finally, differences between correlations in the ASD suggest that the relationship between sleep and daytime cognitive functioning takes different brain routes or altered connectivity substrates in these two populations [35,36,37,38,39,40].

The present results disclose that objective signs of poor sleep in persons with ASD are associated with some limitations in performance. This confirms that it is not sleep quantity but sleep quality that is the determining factor, as suggested by Brett [30] and others [33,34,35,36,41].

Study limitations: In addition, our sample of cognitive tasks was based on predicted sleep-cognitive relationships previously reported in typical individuals. Therefore, sleep-daytime performance relationships specific to autism may have been overlooked, including other cognitive functions, such as flexibility of attention. Also, sample size, which corresponded to current practice in clinical research, may still have been insufficient to unravel subtle effects. Finally, this study included a large number of correlation analyses so that the findings need to be replicated with more participants and appropriate statistical procedures to reduce the risk of type 1 and type 2 errors.

Conclusion: In summary, our results suggest that reports of sleep-onset delay on the CSHQ were more closely related to the objective measurements and correlated with delay in response time of reaction time task. This conclusion is still premature and requires further investigation for more detail study. Since sleep is a central mechanism for adaptive functioning (e.g., learning, memory, brain plasticity), it is highly reasonable that sleep deficits play a leading role in the symptoms seen in ASD including the less social interaction, limited communication, and a restricted repertoire of behaviors, interests, and activities. Further studies exploring the causal mechanisms of insomnia in children with ASD, as well as intervention studies aimed at improving their sleep through behavior, pharmacologic, and integrative modalities, is warranted. Research for the etiology and treatment of sleep disturbances in children with ASD is urgently needed. Defining the phenotype of sleep in ASD, its relation to daytime behavior, and appropriate measurement modalities provides the foundation for focused studies of sleep pathophysiology and cognitive function in this population.

## Figures and Tables

**Table 1:** Comparison of Children’s Sleep Habits Questionnaire (CSHQ) subscales and actigraphy variable among autism spectrum disorder (ASD) Poor Sleepers (ASD-PS), ASD Good Sleepers (ASD-GS).

Assessment Tools	Variable	ASD Group	Mean ± Std. D	*p* Value
	Age	ASD-PS	9.4 ± 1.13	0.622
		ASD-GS	9.2 ± 1.05	
CSHQ	Bedtime_Resistance	ASD-PS	7.7 ± 0.64	0.001
		ASD-GS	6.3 ± 0.92	
	Sleep_Duration	ASD-PS	4.4 ± 0.67	0.021
		ASD-GS	3.6 ± 0.70	
	Sleep_Anxiety	ASD-PS	5.7 ± 0.60	0.019
		ASD-GS	5.03 ± 0.52	
	NightWakings	ASD-PS	5.3 ± 0.51	0.007
		ASD-GS	4.4 ± 0.68	
	Total score	ASD-PS	47.8 ± 3.0	0.001
		ASD-GS	52.8 ± 2.2	
Actigraphy	Total Sleep Time (TST)	ASD-PS	433.9 ± 26.02	0.083
		ASD-GS	455.9 ± 24.05	
	Efficiency	ASD-PS	66.6 ± 9.13	0.189
		ASD-GS	72.6 ± 9.3	
	Wake After Sleep Onset (WASO)	ASD-PS	66.7 ± 9.18	0.077
		ASD-GS	59.03 ± 7.6	
	Number of Awakenings	ASD-PS	22.2 ± 5.2	0.692
		ASD-GS	21.4 ± 2.3	

**Table 2:** Comparison of Cambridge Neuropsychological Test Automated Battery (CANTAB) tasks including, intradimensional/extradimensional shift (IED) motor planning task (MOT), simple reaction time task (SRT), among ASD Poor Sleepers (ASD-PS), ASD Good Sleepers (ASD-GS).

CANTAB Variable	ASD Group	Mean ± Std. D	Min-Max	*p* Value
IED	ASD-PS	44.3 ± 18.8	12.00–63.00	0.789
	ASD-GS	41.6 ± 22.9	11.00–63.00	
IEDStages	ASD-PS	8.0 ± 1.0	7.00–9.00	0.801
	ASD-GS	8.1 ± 0.9	7.00–9.00	
MOT	ASD-PS	931.4 ± 281.9	559.1–1394.0	0.496
	ASD-GS	853.5 ± 158.4	703.7–1204.5	
MOT_Error	ASD-PS	12.2 ± 2.0	9.01–15.7	0.193
	ASD-GS	13.4 ± 1.5	11.7–15.7	
SRT	ASD-PS	806.9 ± 50.6	740.7–900.0	0.035
	ASD-GS	759.8 ± 30.1	699.0–785.4	
SRTMaximum	ASD-PS	799.8 ± 106.8	698.5–998.0	0.130
	ASD-GS	737.6 ± 27.6	709.5–782.5	
SRT_SD	ASD-PS	464.4 ± 238.4	250.5–716.5	0.092
	ASD-GS	631.9 ± 125.2	553.3–920.4	
SRT_Percentage	ASD-PS	96.2 ± 2.3	92.0–100	0.386
	ASD-GS	97.5 ± 3.5	92.0–100	

## References

[1] American Psychiatric Association (2013). Diagnostic and Statistical Manual of Mental Disorders (DSM-V) 5.

[2] Krakowiak P., Goodlin-Jones B., Hertz-Picciotto I., Croen L.A., Hansen R.L. (2008). Sleep problems in children with autism spectrum disorders, developmental delays, and typical development: A population-based study. J. Sleep Res..

[3] Couturier J.L., Speechley K.N., Steele M., Norman R., Stringer B., Nicolson R. (2005). Parental perception of sleep problems in children of normal intelligence with pervasive developmental disorders: Prevalence, severity, and pattern. J. Am. Acad. Child Adolesc. Psychiatry.

[4] Owens J.A., Spirito A., McGuinn M. (2000). The Children’s Sleep Habits Questionnaire (CSHQ): Psychometric properties of a survey instrument for school-aged children. Sleep.

[5] Malow B.A., Marzec M., McGrew S.G., Wang L., Stone W. (2006). Characterizing sleep in children with autism spectrum disorders: A multidimensional approach. Sleep.

[6] Goodlin-Jones B.L., Sitnick S.L., Tang K., Liu J., Anders T.F. (2008). The children’s sleep habits questionnaire in toddlers and preschool children. J. Dev. Behav. Pediatr..

[7] Sitnick S.L., Goodlin-Jones B.L., Anders T.F. (2008). The use of actigraphy to study sleep disorders in preschoolers: Some concerns about detection of nighttime awakenings. Sleep.

[8] Wiggs L., Stores G. (2004). Sleep patterns and sleep disorders in children with autistic spectrum disorders: Insights using parent report and actigraphy. Dev. Med. Child Neurol..

[9] Goldstein G., Johnson C.R., Minshew N.J. (2001). Attentional processes in autism. J. Autism Dev. Disord..

[10] Elia M., Ferri R., Musumeci S.A., Del Gracco S., Bottitta M., Scuderi C., Miano G., Panerai S., Bertrand T., Grubar J.C. (2000). Sleep in subjects with autistic disorder: A neurophysiological study. Brain Dev..

[11] Miano S., Ferri R. (2010). Epidemiology and management of insomnia in children with autistic spectrum disorders. Paediatr. Drugs.

[12] Schreck K.A., Mulick J.A. (2000). Parental reports of sleep problems in children with autism. J. Autism Dev. Disord..

[13] Sadeh A., Pergamin L., Bar-Haim Y. (2006). Sleep in children with attention deficit hyperactivity disorders: A meta-analysis of polysomnographic studies. Sleep Med. Rev..

[14] Golombek D.A., Casiraghi L.P., Agostino P.V., Paladino N., Duhart J.M., Plano S.A., Chiesa J.J. (2013). The times they’re a-changing: Effects of circadian desynchronization on physiology disease. J. Physiol. Paris.

[15] Touchette E., Côté S.M., Petit D., Liu X., Boivin M., Falissard B., Tremblay R.E., Montplaisir J.Y. (2009). Short nighttime sleep-duration and hyperactivity trajectories in early childhood. Pediatrics.

[16] Johnson K.P., Giannotti F., Cortesi F. (2009). Sleep patterns in autism spectrum disorders. Child Adolesc. Psychiatr. Clin. N. Am..

[17] Schwichtenberg A.J., Young G.S., Hutman T., Iosif A.M., Sigman M., Rogers S.J., Ozonoff S. (2013). Behavior and sleep problems in children with a family history of autism. Autism Res..

[18] Taylor M.A., Schreck K.A., Mulick J.A. (2012). Sleep disruption as a correlate to cognitive and adaptive behavior problems in autism spectrum disorders. Res. Dev. Disabil..

[19] Pascualvaca D.M., Fantie B.D., Papageorgiou M., Mirsky A.F. (1998). Attentional capacities in children with autism: Is there a general deficit in shifting focus?. J. Autism Dev. Disord..

[20] Belleville S., Menard E., Mottron L., Menard M.C., Allowa T.P., Gathercole S. (2006). Working memory in autism. Working Memory and Neurodevelopmental Disorders.

[21] Geurts H.M., Verte S., Oosterlaan J., Roeyers H., Sergeant J.A. (2004). How specific are executive functioning deficits in attention deficit hyperactivity disorder and autism?. J. Child Psychol. Psychiatry.

[22] Minshew N.J., Luna B., Sweeney J.A. (1999). Oculomotor evidence for neocortical systems but not cerebellar dysfunction in autism. Neurology.

[23] Mostofsky S.H., Goldberg M.C., Landa R.J., Denckla M.B. (2000). Evidence for a deficit in procedural learning in children and adolescents with autism: Implications for cerebellar contribution. J. Int. Neuropsychol. Soc..

[24] Luciana M. (2003). Practitioner review: Computerized assessment of neuropsychological function in children: Clinical and research applications of the Cambridge Neuropsychological Testing Automated Battery (CANTAB). J. Child Psychol. Psychiatry.

[25] Open Science Collaboration (2015). Estimating the reproducibility of psychological science. Science.

[26] Sahakian B.J., Owen A.M. (1992). Computerized assessment in neuropsychiatry using CANTAB: Discussion paper. J. R. Soc. Med..

[27] Smith P.J., Need A.C., Cirulli E.T., Chiba-Falek O., Attix D.K. (2013). A comparison of the Cambridge Automated Neuropsychological Test Battery (CANTAB) with “traditional” neuropsychological testing instruments. J. Clin. Exp. Neuropsychol..

[28] De Rover M., Pironti V.A., McCabe J.A., Acosta-Cabronero J., Arana F.S., Morein-Zamir S., Hodges J.R., Robbins T.W., Fletcher P.C., Nestor P.J. (2011). Hippocampal dysfunction in patients with mild cognitive impairment: A functional neuroimaging study of a visuospatial paired associates learning task. Neuropsychologia.

[29] Robbins T.W., James M., Owen A.M., Sahakian B.J., Lawrence A.D., McInnes L., Rabbitt P.M. (1998). A study of performance on tests from the CANTAB battery sensitive to frontal lobe dysfunction in a large sample of normal volunteers: Implications for theories of executive functioning and cognitive aging. Cambridge Neuropsychological Test Automated Battery. J. Int. Neuropsychol. Soc..

[30] De Luca C.R., Wood S.J., Anderson V., Buchanan J.A., Proffitt T.M., Mahony K., Pantelis C. (2003). Normative data from the CANTAB. I: Development of executive function over the lifespan. J. Clin. Exp. Neuropsychol..

[31] Brett Z.H., Humphreys K.L., Fleming A.S., Kraemer G.W., Drury S.S. (2015). Using crossspecies comparisons and neurobiological framework to understand earlysocial deprivation effects on behavioral development. Dev. Psychopathol..

[32] Bogaczewicz A., Sobow T., Kowalski J., Ząbek J., Woźniacka A., Bogaczewicz J. (2015). Cambridge Neuropsychological Test Automated Battery in assessment of cognitive parameters in patients with systemic lupus erythematosus in relation to autoantibody profile. Reumatologia.

[33] Baddam S.K.R., Canapari C.A., van Noordt S.J.R., Crowley M.J. (2018). Sleep disturbances in child and adolescent mental health disorders: a review of the variability of objective sleep markers. Med. Sci. (Basel).

[34] Mazzone L., Postorino V., Siracusano M., Riccioni A., Curatolo P. (2018). The Relationship between Sleep Problems, Neurobiological Alterations, Core Symptoms of Autism Spectrum Disorder, and Psychiatric Comorbidities. J. Clin. Med..

[35] Sadeh A., Gruber R., Raviv A. (2002). Sleep, neurobehavioral functioning, and behavior problems in school-age children. Child Dev..

[36] Happe F., Frith U. (1996). The neuropsychology of autism. Brain.

[37] Belmonte M.K., Yurgelun-Todd D.A. (2003). Functional anatomy of impaired selective attention and compensatory processing in autism. Brain Res. Cognit. Brain Res..

[38] Luna B., Minshew N.J., Garver K.E., Lazar N.A., Thulborn K.R., Eddy W.F., Sweeney J.A. (2002). Neocortical system abnormalities in autism: An fMRI study of spatial working memory. Neurology.

[39] Muller R.A., Kleinhans N., Kemmotsu N., Pierce K., Courchesne E. (2003). Abnormal variability and distribution of functional maps in autism: An FMRI study of visuomotor learning. Am. J. Psychiatry.

[40] Giuditta A., Ambrosini M.V., Montagnese P., Mandile P., Cotugno M., Grassi Zucconi G., Vescia S. (1995). The sequential hypothesis of the function of sleep. Behav. Brain Res..

[41] Stickgold R., Whidbee D., Schirmer B., Patel V., Hobson J.A. (2000). Visual discrimination task improvement: A multi-step process occurring during sleep. J. Cognit. Neurosci..

